# Long-term hydrodynamic changes in marginal estuarine seas: the role of sea level rise and freshwater fluxes

**DOI:** 10.1038/s41598-025-33172-7

**Published:** 2025-12-26

**Authors:** Emil V. Stanev

**Affiliations:** 1 Institute of Coastal Systems ‑ Analysis and Modeling, Helmholtz-Zentrum Hereon, Geesthacht, Germany; 2 Department of Meteorology and Geophysics, University of Sofia “St. Kliment Ohridski”, Sofia, Bulgaria

**Keywords:** Climate sciences, Environmental sciences, Hydrology, Ocean sciences

## Abstract

Significant environmental changes have occurred in marginal estuarine seas in the past, driven by changes in sea level and water and salt balances. Predicting the future evolution of these seas is a scientific challenge because adequate scenario studies are currently hindered by the quality of 3D models on millennial timescales. In this paper, we present relatively simple and efficient models of the Black Sea. This sea connects rivers to the ocean and acts as a ‘mixer’, diluting inflowing saline ocean water with riverine freshwater to generate brackish water. For such an ocean system, it is crucial to incorporate mixing within the sea and two-layer exchange within the strait into a single framework. This paper demonstrates that the developed models can be used to (1) estimate past and future ocean states resulting from specific combinations of global sea level and freshwater fluxes; (2) critically address the consistency of used water and salt balances; (3) conceptualizing further studies of major environmental trends. Illustrations are provided to show the evolution of stratification of the Black Sea in the Holocene. Application to other ocean environments controlled by two-layer exchange flows is also discussed.

**Supplementary Information:**

The online version contains supplementary material available at 10.1038/s41598-025-33172-7.

## Introduction

**Fig. 1:**
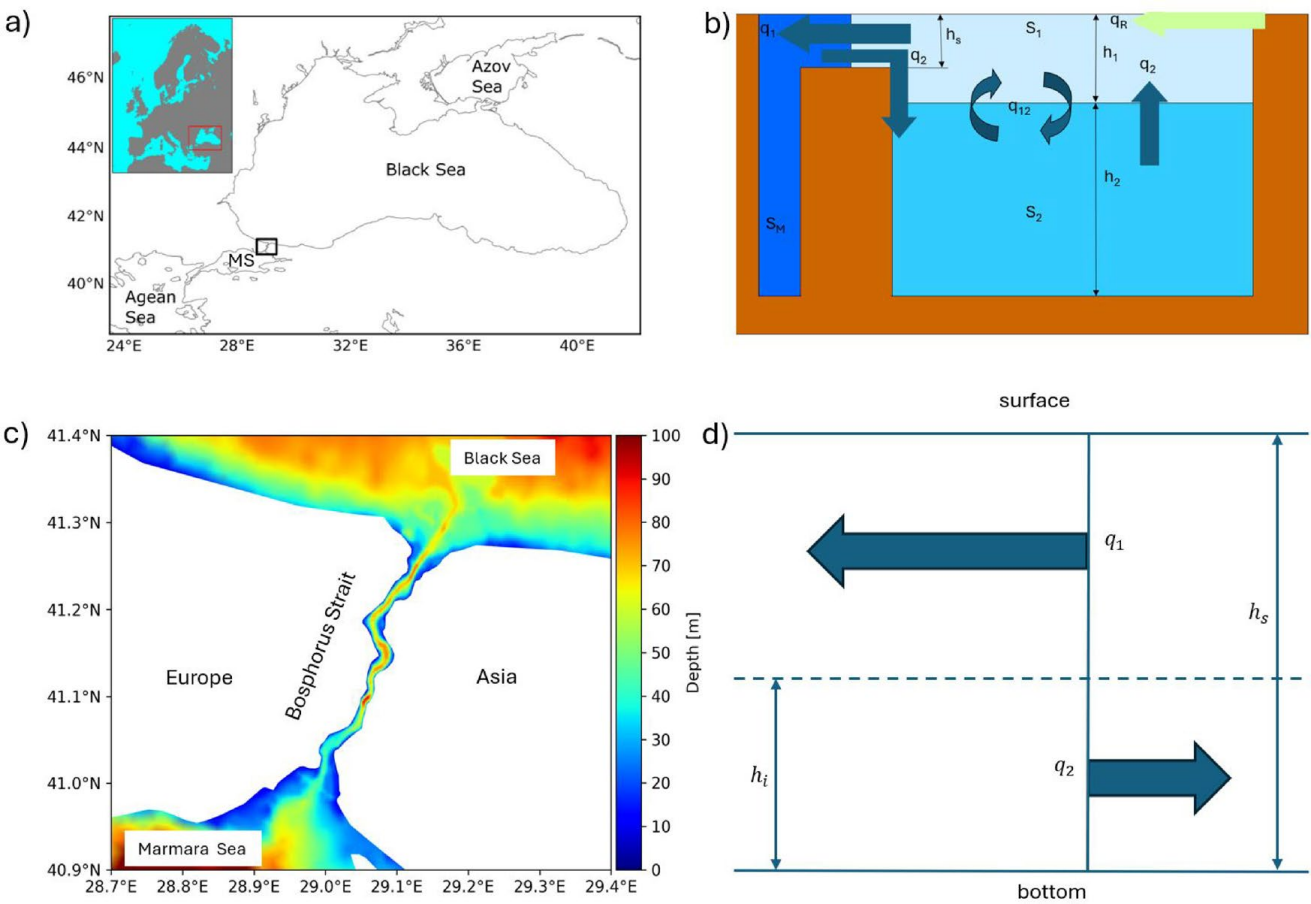
(**a** and **c**) The Black Sea. Its location is shown as an insect in the upper-left corner of (**a**). MS stands for “Marmara Sea”. The magnified bathymetry map in (**c**) is the region of Bosphorus Strait shown in (**a**) as a small black box. (**b**) is a schematic presentation of the box model, and (**d**) shows the exchange flow in the strait. Some of the notations used are also given.

Marginal seas are partially enclosed bodies of water that border continents. They are characterized by limited water exchange with the open ocean. Depending on the water balance, they are either estuarine seas where river runoff and precipitation exceed evaporation (e.g. the Black Sea and the Baltic Sea) or lagoon seas, where the opposite is true (e.g. the Mediterranean and the Red Seas). In this study, we will focus on the Black Sea, an estuarine marginal sea that connects rivers to the ocean via the Strait of Bosphorus (Fig. 1a, c). This strait is shallow and narrow (only ~ 30 m deep and ~ 1 km wide), severely limiting the exchange of water and salt with the Mediterranean Sea. For brevity, we will also use the terms ‘estuarine sea’ or simply ‘sea’ or ‘basin’ throughout the text, bearing in mind that we are referring to a ‘marginal sea of estuarine type’.

The paleo evolution of the Black Sea is an interesting example of long-term environmental transitions in response to changes in global sea level and regional freshwater flux^1^. Many studies focus primarily on the impact of sea level rise, including thermal expansion, on the future climatic states of the ocean. For marginal seas, the effect of freshwater is equally important. However, they have not received much attention in relation to their sensitivity to changes in sea level rise and water fluxes, unlike river estuaries^2,3^.

This study considers sea level rise and freshwater fluxes within the same framework alongside the issue of basin-wide mixing and its relationship with exchange flows through straits. This is a fundamental approach to understanding the dynamics of the Black Sea because (1) this sea acts as a ‘mixer’, diluting the inflowing saline ocean water with riverine water and (2) basin-wide mixing depends on exchange through the Strait of Bosphorus. The latter can be described by the theory of two-layer hydraulics^4–6^, which deals with the density-driven water exchange in narrow passages between two bodies of water. This theory states that the flow in a strait remains unidirectional until the thickness of the water column within the strait increases beyond a critical threshold

1 $$h_{c} = \left( {\frac{{q_{R}^{2} }}{{g\frac{{_{{ \rho_{M} - \rho_{1} }} }}{{\rho_{M} }}W^{2} }}} \right)^{\frac{1}{3}} ,$$

where $q_{R}^{{}}$ is the fresh-water flux (rivers plus precipitation minus evaporation), *g* is the acceleration due to gravity, $\rho_{M}$ and $\rho_{1}$ are the densities of Marmara Sea water and outflowing water respectively, and *W* is the width of the strait. Once the threshold value is exceeded, the flow becomes a hydraulically critical two-layer current. A surface current flows into the Sea of Marmara, while a countercurrent flows into the Black Sea through the bottom layer.

In the past 20,000 yrs, changes in global sea level and regional fresh-water fluxes resulted in significant alterations to water and salt balances^1^. The onset of two-layer exchange contributed to the gradual increase in salinity in the Black Sea^7^, and it has evolved from an enclosed lake that was decoupled from the Mediterranean Sea under sea level low-stand to its current state of a strongly stratified estuarine sea with anoxic conditions dominating the water column below ~ 150 m (Supporting Information SI-1 provides an overview of the evolution of this sea during the Holocene). Equally intriguing is the prospect of what will happen to marginal seas in the future, given their high sensitivity to climate change and human impact. A possible change in the salinity stratification of the Black Sea could result in a substantial change in the volume of anoxic water, or even complete ventilation, as occurred in the past^1^. This makes it an excellent case study to examine the evolution of the exchange flows through the strait and vertical stratification.

The above considerations demonstrate that the state of a marginal estuarine sea is driven by two key parameters: sea level and freshwater flux. The present study aims to investigate the sensitivity of two-layer exchange within the strait in response to changes in these two parameters, and the formation of vertical stratification in the sea. This is of the utmost importance, as the stratification of the Black Sea (and other similar estuarine seas) controls most of the dominant balances in its physical and biogeochemical systems. As global warming progresses, the ocean level is expected to rise. This would affect the balance in the connecting straits: the surface and countercurrents would increase; the influx of salt into the estuarine seas would increase; and the halocline would deepen. The first part of this study quantifies the steady hydrological states resulting from the various combinations of sea levels and freshwater flows. In essence, we apply the analysis of exchange flows in straits^6^ to stratified marginal seas following the idea that the two-layer exchange in the mouth acts as a constraint on the mixing in the estuary^8^. For the first part of our study, we use a box model that takes into account the basic hydrological and salinity balances in the Black Sea, as well as the entrainment of Black Sea water by the Bosphorus plume.

Box models do not fully address the mixing between layers. The convective penetration of gravity plumes is also not adequately represented; the model used in the first part of the present study is stationary. To overcome these limitations, the second part of the present study uses a coupled 1D mixed-layer convective plume model. This model solves evolutionary problems and provides realistic vertical stratification. It can thus replicate the Holocene evolution of exchange flows and stratification for the Black Sea under sea level rise. The usefulness of the new knowledge for making first order estimates of basin states during periods of rapid climate change and for conceptualizing further studies of major environmental trends in marginal estuarine seas will be demonstrated.

## Theory and methodology

### Box model

#### Conservation equations

Here, we consider a two-box model (Fig. 1b). The boxes are connected by a strait of depth $h_{s}$. One of the boxes represents a vertically homogeneous body of water with salinity $S_{M}$ (index “M” stands for Mediterranean). The second box is a two-layer basin, the thicknesses of the upper and lower layers being $h_{1} {\mathrm{and}} h_{2}$, and salinities $S_{1}^{{}}$ and $S_{2}^{{}} ,$ respectively. The thickness of the upper layer, $h_{1} {\mathrm{is}}$ defined as the depth at which the salinity is equal to the vertically mean salinity. Using a representative observed vertical salinity profile, this depth is estimated as $\approx 200 m$. This simplified representation is reminiscent of the coupled Mediterranean and Black Sea system, where the vertical salinity stratification in the Mediterranean Sea is much weaker than in the Black Sea; the latter exhibits a practically two-layer stratification. Keeping in mind that paleo evolution is a very slow process, we will be analyzing the stationary states of the above two-basin system.

In the stationary case, the system of conservation equations for salt and water (see also Supporting information, Eqs. SI-2.1, SI-2.2 and SI-2.3) reduces to

2 $$q_{2} S_{2} - q_{1} S_{1} - q_{12} \left( {S_{1} - S_{2} } \right) = 0$$

3 $$q_{2} \left( {S_{M} - S_{2} } \right) + q_{12} \left( {S_{1} - S_{2} } \right) = 0$$

4 $$q_{1} - q_{2} = q_{R} ,$$

where $q_{1}$ and $q_{2}$ are the flows in the surface and bottom layer in the strait, (Fig. 1d), and $q_{12}$ is the exchange flow between the two layers^9–11^. Summing Eq. (2) and Eq. (3) yields the conservation equation for salt

5 $$q_{1} S_{1 = } q_{2} S_{M}^{{}}$$

It follows from Eqs. (4) and (5) that

6 $$q_{1} = q_{R} \frac{{S_{M} }}{{S_{M} - S_{1} }}$$

7 $$q_{2} = q_{R} \frac{{S_{1} }}{{S_{M} - S_{1} }}$$

Equations (6) and (7) are the Knudsen relationships^12^, which quantify exchange flows, based on the mass conservation laws. Note that the variable $S_{2}$ does not enter the Knudsen relationships, yet these relationships are still valid for a two-layer ocean.

It follows from Eq. (6)

8 $$S_{1} = S_{M} \left( {1 - \frac{{q_{R} }}{{q_{1} }}} \right),$$

which is another form of the Knudsen relationships. The ratio $\frac{{S_{1} }}{{S_{M} }}$ describes the mixing completeness^13^. Salinity in the deep layer can be computed from Eqs. (3 and 5) as

9 $$S_{2} = S_{M} \frac{{1 + \frac{{q_{12} }}{{q_{1} }}}}{{1 + \frac{{q_{12} }}{{q_{2} }}}}$$

Equation (9) shows that the proposed model goes beyond the Knudsen relationships because it considers the exchange flow between layers $q_{12}$ and makes it possible to calculate $S_{2}$. For the ratio between upper- and lower-layer salinities, we can write

10 $$\frac{{S_{1} }}{{S_{2} }} = \frac{{q_{12} + q_{2} }}{{q_{12} + q_{1} }} = \frac{{1 + \frac{{q_{2} }}{{q_{12} }}}}{{1 + \frac{{q_{1} }}{{q_{12} }}}},$$

As seen from Eq. (10), $\frac{{S_{1} }}{{S_{2} }}$ depends on both the transport through the strait ($q_{1} , q_{2}$) and the exchange between layers ($q_{12}$). As $q_{12}$ becomes large, $S_{1} { }$ approaches a similar value as $S_{2}$ creating well-mixed conditions. From Eq. (10), we can express the mixing $q_{12}$ as a function of the salinity of the upper and deep layers, as well as the exchange flows:

11 $$q_{12} = \frac{{q_{2} S_{2} - q_{1} S_{1} }}{{S_{1} - S_{2} }}$$

This equation enables an estimation of $q_{12}$(see section “Application results and discussion)”.

#### Model physics

*The concept of mixing *The mixing model is concerned with the interaction of the Bosphorus plume that carries dense Mediterranean water into the Black Sea through the Strait and the water within the Black Sea. Equations (11 and 5) can be used to estimate the entrainment of Black Sea water by the Bosphorus plume as:

12 $$\frac{{q_{12} }}{{q_{2} }} = \frac{{S_{M} - S_{2} }}{{S_{2} - S_{1} }} = E,$$

which gives an overall measure of the mixing of waters from the Bosphorus plume and ambient waters. According to Eq. (12), in the case of extremely strong stratification ($S_{2} {\text{ tends to }}S_{M}$), $q_{12} {\text{ tends to }}0$. Conversely, in the case of weak stratification ($S_{2} {\text{tends to }}S_{1}$), the exchange between layers is large ($q_{12} > > q_{2}$), enabling mixing due to mechanical energy to penetrate deep.

Laboratory experiments^14^, as well as dimensional considerations^15^ concerning mixing in the upper ocean, led to the concept that the rate of increase of the potential energy of the stratified fluid is proportional to the rate at which kinetic energy is dissipated. The following equation for the entrainment was proposed^14^

13 $${\mathrm{E}} = \frac{{u_{e} }}{{v_{*} }} = 2.5{ }Ri^{ - 1} = 2.5{ }\frac{{\rho v_{*}^{2} }}{g\Delta \rho D}\sim { }\frac{{E_{{\mathrm{k}}}^{{}} }}{{E_{{\mathrm{p}}} }},$$

where $u_{e}$ and $v_{*}$ are the entrainment and friction velocity $(v_{*} = \sqrt {\frac{\tau }{\rho }}$ ), $Ri$ is the gradient Richardson number, $\rho$ is the density, $\Delta \rho$ is the density difference between layers, and $D$ is the thickness of the upper mixed layer. $E$ is a measure of the relative magnitudes of the effects produced by wind and buoyancy. We must acknowledge that Eq. (13) is not strictly applicable to our case, as upper ocean mixing does not fully describe mixing down to the depth of the halocline. Therefore, the $E_{{\mathrm{k}}}^{{}}$ term above should be considered as resulting from mixing that penetrates deeper than the base of the mixed layer (the concept of Black Sea mixing is presented in more detail in Supporting information SI-3). In summary, the influx of surface freshwater and inflow from the strait maintain the stability of stratification. Mixing (e.g. associated with wind-driven circulation) tends to deepen the halocline by entraining deep water into the surface layer, thereby opposing the dilution of surface waters by rivers. Furthermore, the Bosphorus plume does not simply propagate through a predefined ambient water column; rather, the water column itself adjusts to the processes that govern the plume’s propagation.

By formally replacing $v_{*}^{2}$ by $v_{{{\mathrm{Sv}}}}^{2} ,$ where $v_{{{\mathrm{Sv}}}}$ is a characteristic velocity for the wind-driven circulation (e.g. associated with the wind stress curl), and assuming that density is linearly dependent on salinity, it follows from Eqs. (12 and 13) that

14 $$\left( {\rho_{M} - \rho_{2} } \right) \cdot h_{1} = 2.5\frac{{\rho v_{{{\mathrm{Sv}}}}^{2} }}{g}$$

This means that, for a fixed external driver (e.g. annual mean wind stress curl), the product on the left-hand side of Eq. (14) remains constant. Assuming that this external driver does not substantially change in the long term, the above equation can be used to calibrate the model against the known present-day values of $\rho_{2 }$ and $h_{1}$. If $\rho_{M }$ does not change substantially over long periods of time— which is plausible given that the Mediterranean remained coupled to the Atlantic Ocean —then it follows that

15 $$h_{1} = h_{1}^{pr} \frac{{\rho_{M}^{pr} - \rho_{2}^{pr} }}{{\rho_{M} - \rho_{2} }} \approx h_{1}^{pr} \frac{{S_{M}^{pr} - S_{2}^{pr} }}{{S_{M} - S_{2} }} = h_{1}^{pr} \frac{{1 - S_{2}^{pr} /S_{M} }}{{1 - S_{2}^{{}} /S_{M} }},$$

where the index "*pr*" stands for present-day and $S_{M}$ can be considered as constant over time.

*Strait exchange *Now the problem is to know $q_{1}$ , $q_{2}$ and $q_{12}$ in order to compute $S_{1}$, $S_{2}$, $h_{1}$, ${\text{and }}h_{2}$. We remember that most of the above considerations were based on the conservation of salt and mass (Eqs. 2–4), as well as the concept of entrainment (Eqs. 12, 13). The basic physical considerations below originate from the theory of two-layer hydraulics^4,5^, which represents straits flow relatively well. The dynamics in the strait are primarily controlled by the density difference between basins (Eq. 1), and the transport in the upper layer can be computed as^6^

16 $$q_{1} = q_{R} \frac{1 + a}{b},$$

where

17 $$a = \left( {1 - bc} \right)^{\frac{1}{2}} ,{ }b = 1 + \left( {\frac{{h_{i} }}{{h_{s} - h_{i} }}} \right)^{3} ,{ }c = 1 - \left( {\frac{{h_{i} }}{{h_{c} }}} \right)^{3}$$

In Eq. (17), $h_{s}$ is the total depth above the sill; in other words, it measures the sea level height and

18 $$h_{i} = \frac{{h_{s} }}{2}\left( {1 - (\frac{{h_{c} }}{h}} \right)^{\frac{3}{2}} )$$

is the height of the interface above the sill (a brief explanation of the two-layer hydraulics is given in the Introduction). This equation, along with Eq. (6), forms a closed system of equations.

### Coupled mixed layer-convective plume model

As a step beyond the box-model, we can use the 1D coupled mixed layer-convective plume model to compute vertical stratification. We will only provide a brief introduction to the model we use here, as it was described in detail earlier^16^. It consists of two parts: the open ocean column and the Bosphorus plume, which carries dense Mediterranean water into the deep ocean layers (see also supporting information SI-4). Physics of the ocean column includes the parameterization of the surface mixed layer and vertical mixing in the deeper layers, where the diffusion coefficient depends on stability of stratification. The plume model ensures that, at any depth, the plume acquires Black Sea water characteristics through entrainment and exports plume water (of higher salinity) into the model column. The evolution of the plume adheres to the theories of buoyant plumes^15,17–19^, atmospheric modelling^20^, and the results of laboratory and theoretical studies^21,22^. The coupled mixed layer-convective plume model has been developed as a module to be integrated into the 3D Modular Ocean Model (MOM)^16,23^. This module operates formally in a similar way to the convective adjustment in the 3D model. Thus, the convective element does not directly affect momentum equations. Calibrating entrainment based on Eq. (12) ensures the realism of simulations. The performance of the model and the quantification of stratification sensitivity to entrainment formulation have also been analyzed previously^16^.

The mixed layer part of the water-column model relates changes in the potential energy of a vertical water column to the wind’s working rate at the sea surface^24^. The advantage of the 1D model over the box model is that the former provides continuous vertical stratification as an output and simulates temporal evolution. Its disadvantage is that a great number of individual experiments need to be carried out to test their sensitivity to external drivers.

## Model applications

### Steady-state exchange flows and freshwater distribution

Equations (17 and 6) can be solved iteratively for $q_{1}$ and $S_{1}$ within certain ranges of $q_{R}$ and $h_{s}$ ^25^. Knowing $q_{1}$, one can compute $q_{2}$ from Eq. (4) and use Eq. (12) to compute $q_{12}$. We can then use the balance equations to calculate the diagnostic variables, and further to estimate the fresh-water content defined as

19 $$F_{w} = H - \frac{{h_{1} S_{1} + h_{2} S_{2} }}{{S_{M} }} = h_{1} \left( {1 - \frac{{S_{1} }}{{S_{M} }}} \right) + h_{2} \left( {1 - \frac{{S_{2} }}{{S_{M} }}} \right),$$

where $H = h_{1} + h_{2}$ is total ocean depth. The present-day parameters used in the model are presented in Table 1. This table also provides the model-derived fundamental quantities, such as mixing completeness, exchange between layers, and entrainment. Noteworthy is the rate of entrainment, which agrees well with earlier estimates^10,16^, but disagrees with some others^26^.

**Table 1:** Parameters in the box model corresponding to the present-day Black Sea conditions (see more explanations in section “Box model”). Italicized values are derived from the model, while others were defined as input.

Parameter	Value
Thickness of surface layer	h_1_ = 200 m
Thickness of deep layer	h_2_ = 1800 m
Sill depth	h = 30 m
Width of the Strait	W = 950 m
Mediterranean water salinity	S_m_ = 36
Salinity of surface layer	S_1_ = 18
Salinity of deep layer	S_2_ = 22
Precipitation plus river runoff minus evaporation	$q_{2} = 10^{4} \frac{{{\mathrm{m}}^{3} }}{{\mathrm{s}}}$
Transport in the strait (upper current)^27^	$q_{1} = 2{\mathrm{x}}10^{4} \frac{{{\mathrm{m}}^{3} }}{{\mathrm{s}}}$
Transport in the strait (bottom current)^27^	$q_{2} = 10^{4} \frac{{{\mathrm{m}}^{3} }}{{\mathrm{s}}}$
*Mixing completenes*^13^ (Eq. 8)	$\frac{{S_{1} }}{{S_{M} }} = 0.5$
*Exchange between surface and deep layer* (Eq. 11)	$q_{12} = 3.5x10^{4} \frac{{m^{3} }}{s}$
*Entrainment* (Eq. 12)	E = 3.5

**Fig. 2:**
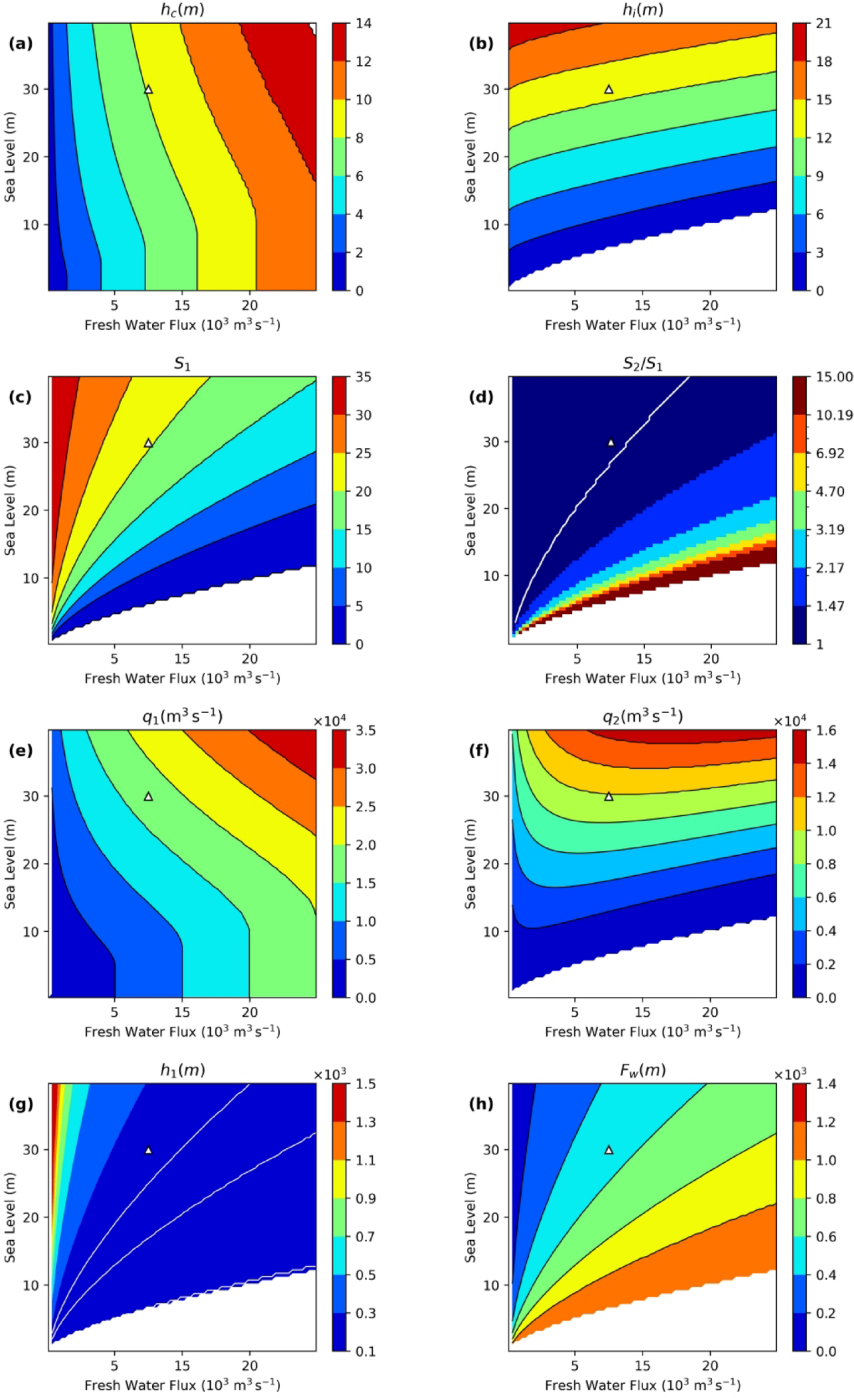
Dependence of parameters of box model on the sea level and fresh-water flux. Panels (**a**)–(**f**) illustrate conditions in the strait, as follows: the critical thickness of the water column $h_{c} ,$ (**a**), interface depth $h_{i}$, (**b**), salinity of the surface current $S_{1} ,$(**c**), the ratio of the salinities of the bottom and surface currents $\frac{{S_{2} }}{{S_{1} }},$ (**d**), the transport by surface current $q_{1}$ (**e**), the bottom current ${\text{transport }}q_{2}$ (**f**). The following two panels show the upper ocean layer thickness $h_{1}$ (**g**), and the amount of fresh water (**h**). White line in (**d**) is 1.2, that is approximately the present-day ratio between salinities of two layers, white lines in (**g**) are isolines 100, 150 and 200 m; triangle symbols correspond to the present-day values of sea level and fresh-water flux.

The solution of the box model is presented below for sea-level ranges above the sill $h_{s}$ from 0 to 40 m, and for a freshwater flux $q_{1}$ from 0 to 20 × $10^{3} \frac{{m^{3} }}{s}$. The main parameter controlling the model solution $h_{c}$ (Eq. 1) is shown in Fig. 2a. Until $h_{s}$ < $h_{c}$, $\rho_{1} = 0$, and $h_{c}$ increases as a function of $q_{R}^{{}}$ only (the flow through the strait is one-directional, like a river). Beyond the critical value ($h_{s}$ > $h_{c}$), a two-layer exchange occurs. With the rising sea level, $h_{c}$ increases because the reduced gravity tends to decrease ($\rho_{1}$ in Eq. 1 increases).

The dependence of $h_{c}$ on the flux of freshwater and the height of the sea level results in a change in the depth of the interface in the strait $h_{i}$, as illustrated in Fig. 2b (see also Eq. 18). Rising sea level tends to raise the interface, while increased freshwater flow tends to lower it. Clearly, the two forces act in opposite directions. A critical situation occurs when the river discharge is sufficiently high, and the sea level is low. In this case, the interface reaches the sill depth, and the flow becomes unidirectional. At this point, only fresh water flows out of the basin (see Fig. 2c).

Depending on the combination of the sea level height above the sill and the freshwater flux, the salinity of the outflow ranges from zero in the case of a unidirectional flow (see bottom part of Fig. 2c) to $S_{M}$ in the case of a high sea level and low fresh-water flux (upper right corner of Fig. 2c). Taking $q_{R}^{{}}$ = $10^{4} \frac{{m^{3} }}{s}$ and *h* = 30 m (present-day conditions), and with a chosen strait width $W = 950{\text{ m}}, S_{1}$ = 17.9 and $S_{2}$ = 21.9 are obtained. These values are approximately equal to the “canonical” values of 18 and 22 (Table 1) that were used when addressing the entrainment, indicating that the model is well calibrated. This suggests that, given the formulated assumptions, there is credibility in the adequacy of the practically infinite number of states shown in Fig. 2.

When $h_{s}$ ≈ $h_{c}$, the salinity of the surface layer approaches zero and the ratio between the two salinities becomes infinite (see Fig. 2d), that is no mixing between layers. This diagram shows that $S_{2} /S_{1}$ ≈1.2 for the present-day conditions.

The evolution of the salinity ratio between the two layers can be easily understood by analyzing the flows in the strait. For $h_{s}$ < $h_{c}$ the upper-layer transport is equal to the fresh-water flux (vertical isolines in Fig. 2e). In other words, the strait acts like a river. Beyond this critical value, upper-layer flow increases as both $h_{s}$ and $q_{R}^{{}}$ increase. The pattern of lower-layer transport (Fig. 2f) is quite different; compare Eqs. (6 and 7). For small $q_{R}^{{}}$ values, $q_{2}^{{}}$ increases with increasing $q_{R}^{{}}$; the trend reverses for large $q_{R}^{{}}$ values.

As can be seen in Fig. 2g, the hydrodynamics of the strait determine the mixing in the ocean water column. The thickness of the upper layer varies widely depending on the flux of fresh water and the position of the sea level. When the freshwater flux is small and the sea level is high, the fresh water occupies only a small volume of the sea (Fig. 2h). In other words, the oceanographic conditions in the marginal estuarine sea resemble those in the open ocean. On the contrary, when the thickness of the water column in the strait approaches the critical depth $h_{c}$, the water column in the marginal sea consists almost entirely of fresh water, that is the sea resembles a river flowing out of a lake.

The model shows a strong dependence on the width of the strait $W$ (Supporting information, Table SI-5.1). This can easily be expected given that the width of the strait and the sill depth are the main geometric parameters of the model that control two-layer exchange (see Eq. 1). Overall, an increase in $W$ tends to increase $S_{1}$ and $S_{2}$, while the difference between $S_{1}$ and $S_{2}$ decreases, as does the ratio between them. Increasing W from 500 to 1,500 m results in a 1.7-fold increase in the upper-layer current and a 3.4-fold increase in the countercurrent. Such an increase in $W$ reduces the fresh-water content in the sea by almost twofold, demonstrating the importance of the straits’ bathymetry for the ocean state. We remind that the results presented above use $W$ = 950 m that is close to the width of the Bosphorus Strait and provides results which are consistent with the widely accepted data on inter-basin exchange^27^.

## Sensitivity of vertical stratification and Holocene evolution

In the current 1D implementation of the coupled mixed layer-convective plume model, the water column is resolved using 24 non-equidistant vertical levels extending down to 2125m^16^. Time stepping is hourly. The meteorological forcing is based on present day monthly wind stress, heat and water fluxes derived from the ECMWF reanalysis data for the Black Sea^16^. Two types of experiment are presented below. The first type primarily addresses the sensitivity of the salinity profile to the freshwater fluxes. The second type aims to simulate the evolution of stratification during Holocene. More specifically, it addresses the gradual reconnection scenario (see supporting information SI-1). For this experiment, sea level evolution is derived from the curve presented in Fig. SI-1.1 and the river runoff is set as the present day one due to a lack of suitable data in the past.

### Sensitivity with respect to freshwater fluxes and sea level

To study the sensitivity with respect to freshwater fluxes, we conducted four experiments. The first one, which is called the Control Run (CR) experiment, is driven by present-day atmospheric and river fluxes and sea level. In this experiment, the sill depth is 30 m. In the sensitivity experiment, called Low River Runoff (LRR) experiment, we used a surface freshwater flux that was half the size of that used in the CR experiment. The next experiment, in which river runoff is double that of the present day, is called the Enhanced River Runoff (ERR) experiment. In the final experiment, we use present-day freshwater fluxes but reduce the sill depth by half.

**Fig. 3:**
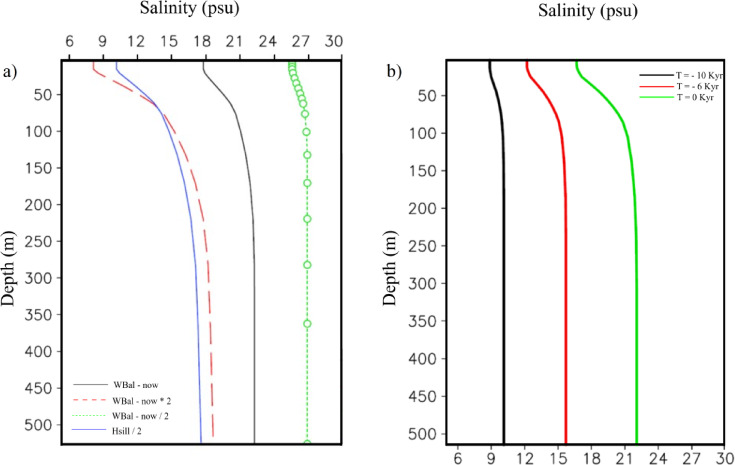
(**a**) Vertical salinity stratification depending on the freshwater balance and sill depth. (**b**) Paleoevolution of the Black Sea salinity illustrated by the vertical salinity stratification at different times prior to the present.

The vertical salinity profiles in the above scenarios (Fig. 3a) show that the sea surface salinity in the ERR and LRR experiments is 10.5 and 25, respectively. In these two experiments, the deep current transport is approximately 0.68 and 1.7 the present-day transport, respectively. These results show that strengthening the two-layer exchange tends to increase the salinity and weaken the vertical stratification.

Stronger stratification in the experiment with an enhanced fresh-water flux results in shallower penetration depth of the Bosphorus plume, and vice versa in the experiment with a reduced fresh-water flux. The penetration depth estimated for the present-day conditions is ~ 500 m, which agrees with the results from observations^10,28^ and numerical modelling^16^. Results from the experiment with a reduced sill depth (the blue line in Fig. 3a) demonstrate that surface salinity decreases substantially (down to 9.8 compared to 17.5 in the CR). Our scenarios clearly demonstrated that the vertical salinity profile of the experiment with the present-day water balance and reduced sill depth resembles that of the experiment with a strong freshwater flux and the present-day sill depth (compare the blue and the red lines in Fig. 3a). An explanation of this result is given in Fig. 2c, which shows that doubling freshwater flux has almost the same effect as a twofold decrease in sill depth.

### Holocene evolution

We conducted an additional experiment to analyze how the Black Sea responds to long-term changes in the water balance. In this experiment, the forcing included a temporally varying sill depth, which was compiled from global ocean sea level paleo data (see upper left panel in Fig. SI-1.1 from the Supporting information). Integration started at 10 ky BP, revealing a monotonic increase in surface salinity from 8 psu at the time of reconnection to 17 psu today. The vertical salinity profiles for three periods (10 ky BP, 6 ky BP, and the present-day situation) are shown in Fig. 3b. Deep-water salinity ranges from 9 to 22 psu over the last 10 ky (16 psu at 6 ky BP).

## Discussion

In line with the main research objective outlined in the introduction, we have developed relatively simple models considering sea level rise and freshwater fluxes within the same theoretical framework. This framework, which incorporates two-layer exchange in straits and basin-wide mixing within one model, enabled us to quantify the sensitivity of the two-layer exchange to changes in these two governing parameters. Using the box model, which obeys conservation laws and adequate physics, we estimated the ratio between the flow in the bottom layer of the strait and the exchange between the surface and deep waters of the Black Sea. This fundamental value of 3.5 determines the overall mixing rate and thus the strength of the vertical salinity stratification.

The proposed models can be considered useful tools for making first-order estimates of oceanographic states during periods of sea-level change and change in freshwater fluxes. They demonstrated the extent to which these two forcing factors could lead to a complete transformation of the ocean state. Regarding the ‘competition’ between these two drivers, the box model analysis demonstrated that comparable changes in the oceanographic states could occur for comparable relative changes in sea level and freshwater fluxes, but with opposing signs. Given the recent rapid changes of precipitation regimes over the Danube River Basin^29,30^, it seems plausible that future changes in freshwater supply to the sea will be relatively greater than relative changes in sea level. Therefore, under this assumption, changes in the state of the Black Sea in the near future are expected to be mainly the result of changes in freshwater flows.

The available estimates of water and salt balances in the Black Sea vary significantly but this issue has not yet been critically addressed. The upper- and lower-layer volume fluxes at the northern exit of the strait were reported to be 375 and 253 km^3^/yr, respectively^31^. However, the ratio between these two transport values, as well as similar estimates for other transects observed by the same authors, is inconsistent with the ~ 2:1 ratio between Marmara Sea and Black Sea salinities, (see Eq. 5), suggesting a problem with the salt balance. Based on 10 years of monthly measurements upper (lower) layer fluxes were estimated as 404 (250) km^3^/yr at the northern end of the strait^32^. In this case, the ratio of outflow to inflow is closer to 2:1, indicating a more reasonable salt balance. However, the magnitude of these flows suggests that the freshwater flux is only ~ 2/3 of the commonly accepted values^27^. Other widely cited estimates of freshwater flux are 7.5 × 10^3^ m^3^/s ^33^ and 13 × 10^3^ m^3^/s^34^. Using the parameters specified in the box model and a freshwater flux of 6.7 × 10^3^ m^3^/s and 7.5 × 10^3^ m^3^/s, we obtain quite different water and salt balances, as well as different stratification in the Black Sea (see Table 2). In view of global warming, one might expect increasing evaporation and decreasing river runoff to reduce the flux of freshwater to the low values mentioned above. This would explain the trends observed in the deep water masses^28,35^.

**Table 2:** Hydrological state for three different fresh-water fluxes. The numbers 2/3 and 3/4 correspond to data^32,33^, respectively. The number 1 corresponds to the estimates^27^.

Parts of present-day run off	$S_{1}$	$S_{2}$	$q_{1}$[m^3^/s]	$q_{2}$[m^3^/s]	$F_{w} \left[ {\mathrm{m}} \right]$
2/3	21.6	24.8	16,520	9920	488
3/4	20.5	23.9	17,600	10,100	526
1	17,9	21.9	19,980	9980	610

Nowadays, powerful numerical modelling is limiting the scope for novel studies based on models with reduced complexity. Therefore, it is reasonable to question whether 3D numerical models would be a more appropriate tool with which to address the issues studied here. The high spatial resolution is of the utmost importance, particularly when addressing processes in the straits. Resolving the dynamics of the Bosphorus Strait requires a resolution of several tens of meters, which can be achieved using unstructured grid models^36^. Recent modelling studies^16,36,37^ have demonstrated the strong and complex relationships between the oceanographic conditions on both sides of the strait and the strait exchange. However, current models are too computationally expensive and not sufficiently mature to be run over millennia, which is necessary for studying long-term changes in interconnected basins. A significant issue with long-term simulations is the drift in model states, which depends on, amongst others, the control of straits.

The present study illustrated that models with reduced complexity could play an important role in climate change research. One outcome of the box model and the more complex coupled 1D mixed-layer convective plume model is that they could facilitate more sophisticated studies of the recent evolution of the Black Sea. Experience has shown that circulation models are sensitive to inter-basin exchange (see for more details Supporting information SI-6). Using simpler, more intuitive models could encourage modelers to accurately constrain the water and salt balances in their 3D models and provide them with accessible preliminary data for further research in this area. When tuning the basin-scale model in the strait (in our case, when specifying W), it is essential to carefully analyze water and salt conservation, as well as the level of realism of the simulated Bosphorus plume penetration depth. Models with reduced complexity, such as box models or 1D models incorporating gravity plume physics, could be used as an initial approach. One practical solution for 3D models would be to adjust the transport in two-layer straits to basic conservation balances, possibly based on reduced-complexity models.

We demonstrated that the coupled 1D mixed-layer convective plume model is a useful extension of the box model. It solves evolutionary problems and provides realistic continuous vertical stratification. The model can be used to facilitate our understanding of not only salinity changes, but also other processes dominated by stratification, such as biogeochemical evolution and past and future environmental transitions^1^. Therefore, the model is a promising candidate for coupling with biogeochemical models that cannot be successfully simplified to the level of a two-layer box. Other important issues to consider in the future are: (1) the impact of strait deepening, or constructing new straits^37^, on the ocean state of interconnected basins, (2) the sensitivity of larger-scale or global climatic models to the exchange in narrow underwater straits, (3) developing a similar research framework to other estuarine seas, also to seas with a negative freshwater balance, e.g. the Persian Gulf or the Red Sea. These should form the basis of further studies.

## Conclusions

A box model is proposed for a marginal estuarine sea that is coupled to the ocean via a strait through two-layer exchange. This model goes beyond the Knudsen relationships by elaborating on the concept of mixing between layers in the marginal sea and two-layer exchange in the strait. Sensitivity experiments quantified the contribution of the primary external forces—namely sea level and freshwater flux—to the shaping of basic oceanographic characteristics in the Black Sea. The fact that the developed box model provides estimates that are consistent with those of more complex models lends credibility to the simulated ocean states. The model results showed that, under present-day conditions, the fundamental ratio of water exchanged between layers due to internal mixing to the inflow through the strait is ≈3.5. The model enables us to estimate the dependence of stratification in the sea on external forcing. A more complex evolutionary model involving continuous vertical stratification has been used to quantify long-term oceanographic trends triggered by changes in freshwater flows and sea-level rise (from Holocene to present day). The proposed models could facilitate an understanding of the evolution of salinity and of processes dominated by stratification, such as biogeochemical evolution and environmental transitions in the past, present and future. The issue of consistency in the water and salt balances of the Black Sea, as empirically proposed in the literature, is also critically addressed, along with the possible consequences for numerical modeling. The results of this study could help conceptualize more sophisticated studies of the recent evolution of the Black Sea and other estuarine seas, particularly regarding the consistency in salt and water balances.

## Supplementary Information

Below is the link to the electronic supplementary material.

Supplementary Material 1

## Data Availability

Data and software are freely available from Zenodo: https://zenodo.org/records/15729082. They include a program used to generate results discussed in the present paper and a sample output netcdf file 17 .
