# Supplementary material for: Long-term hydrodynamic changes in marginal estuarine seas: the role of sea level rise and freshwater fluxes
Source: Sci Rep. 2025 Dec 26;15:44637. doi: 10.1038/s41598-025-33172-7 (PMC12749219; doi:10.1038/s41598-025-33172-7)
Supplement: Supplementary file 1 — Supplementary Material 1 [file 41598_2025_33172_MOESM1_ESM.docx]

Supporting Information

Long-term hydrodynamic changes in semi-enclosed estuarine basins: the role of sea level rise and freshwater fluxes

E. V. Stanev^1, 2^

^1^ Institute of Coastal Systems ‑ Analysis and Modeling, Helmholtz-Zentrum Hereon, Geesthacht, Germany

^2^ Department of Meteorology and Geophysics, University of Sofia “St. Kliment Ohridski”, Sofia, Bulgaria

SI-1. The evolution of the Black Sea during the Holocene

During the last glaciation, the Black Sea salinity reached extremely low values^1^, and the sea was transformed into an enclosed lake with aerobic conditions prevailing throughout the entire water column. Paleo coastline observations revealed that the level of the Black Sea during this period was approximately 100 m lower than that of the ocean. This supports the idea that the Mediterranean and Black Seas were hydrodynamically decoupled. While the timing of changes in ocean sea levels is relatively well known (the top-left panel in Fig. SI.1.1), the temporal evolution of the Black Sea level is uncertain^2, 3^. This uncertainty gives rise to two proposed scenarios. According to the first one, the ocean level first reached the sill of the Bosporus Strait, allowing saline ocean water to pour into the Black Sea, filling it within a short period of time^4^ (catastrophic flood scenario, bottom-left panel in Fig. SI-1.1). The scenario of gradual reconnection^5^ suggests that the Black Sea level first rose above the Bosporus Strait sill, causing Black Sea water to spill into the Sea of Marmara (bottom-right panel in Fig. SI-1.1). Outflow from the Black Sea continued for around two thousand years until the ocean level also reached the Bosporus sill. A further thousand years passed with the sea level in the Mediterranean still rising, but the flow through the strait remaining unidirectional at all depths and hydraulically critical^6^. Once the ocean level had reached the height of the Black Sea level, the latter began to rise in concert with the former.

The current state of exchange between the Black Sea and the Marmara Sea is depicted schematically in the top right panel of Fig. SI-1.1, which illustrates the two-layer current in the Bosporus. The figure also shows that the sinking Bosporus water currently does not reach the deep layers of the water column.

The evolutionary model presented in the main text is applicable to the gradual reconnection scenario. Catastrophic scenario would imply that morphological changes have occurred because of erosion caused by high current velocities. This possibility is beyond the scope of the present study.


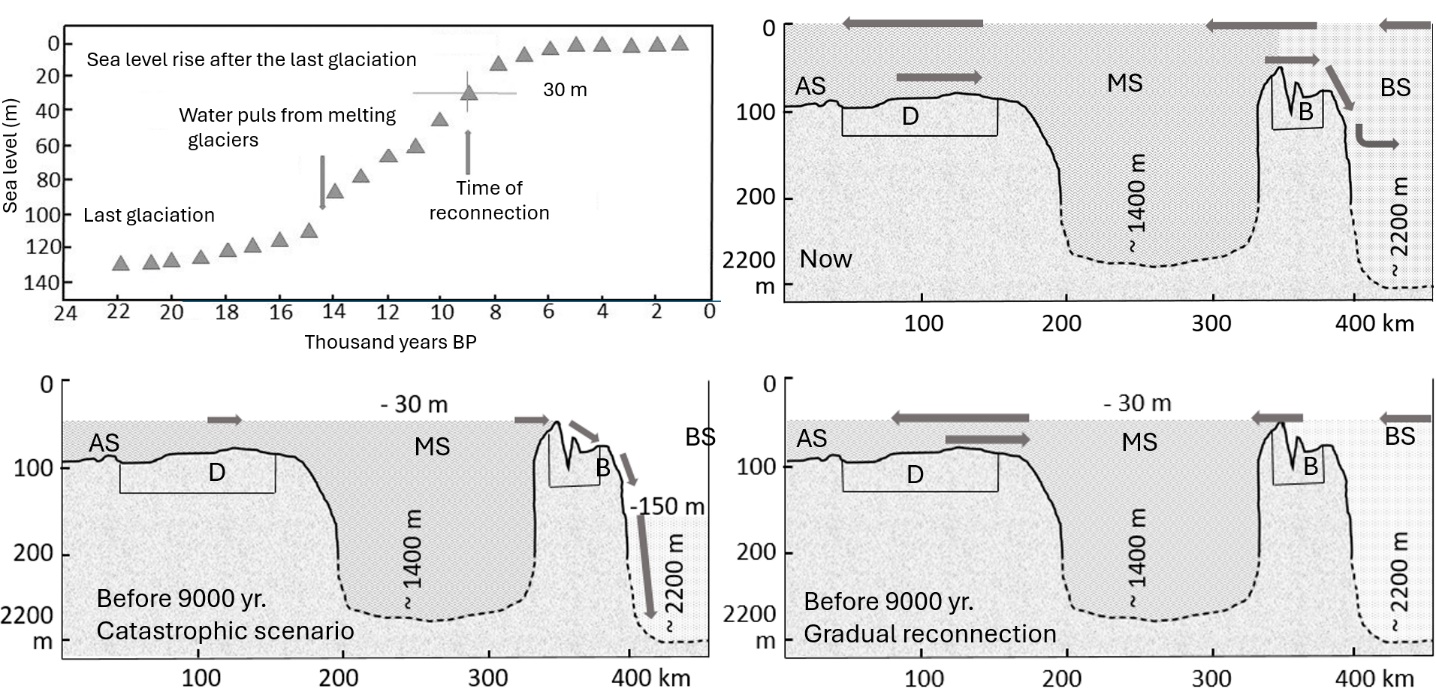


Figure SI-1.1. The top left panel illustrates the rise in the ocean level over the past 25,000 years. The top right panel schematically illustrates the current state of water exchange between the Black Sea and the Mediterranean. The bottom left panel illustrates the catastrophic scenario, while the bottom right panel depicts the gradual reconnection process, which begins with unidirectional flow. The different lengths of the arrows (representing currents) are intended to illustrate the magnitude of the exchange in the straits. The bottom relief of the Sea of Marmara and the straits is also shown schematically. The leftmost and rightmost parts of the diagrams represent adjacent areas of the Black Sea and the Aegean Sea, respectively. Depth (the vertical coordinate) is represented differently in the upper 200 m layer and below. The bathymetry in the deeper region is therefore depicted as a dotted line. The following abbreviations are used: AS: Aegean Sea; MS: Marmara Sea; BS: Black Sea; D: Dardanelles; B: Bosporus. The different shadings reflect the different water characteristics of the individual sea basins. Replotted from^7^.

**SI-2. The conservation equations for salt and water in estuarine basins**

The conservation equations for salt and water in the two-layer estuarine basin can be written as follows (see Fig. 1 and the main text for a description of the model):

$A\frac{d}{dt}S_{1}h_{1}=-q_{12}\left( S_{1}-S_{2} \right)-q_{1}S_{1}+q_{2}S_{2}$ (SI-2.1)

$A\frac{d}{dt}S_{2}h_{2}=q_{12}\left( S_{1}-S_{2} \right)+q_{2}S_{M}-q_{2}S_{2}$ (SI-2.2)

$A\frac{d}{dt}\left( h_{1}+h_{2} \right)=-q_{1}+q_{2}+q_{R},$ (SI-2.3)

where *A* is the basin area, and $q_{12}$ is the exchange flow between the two layers (see also Fig. 1 and the main text for the remaining notations).

**SI-3. The concept of mixing in the Black Sea**

The vertical stratification of Black Sea waters forms as a result of the interplay between two mechanisms: entrainment caused by the Bosporus plume, and Ekman drift of surface waters towards the coast^8^. The latter supports a higher sea level and anticyclonic (downward) circulation in coastal regions. Thus, the sinking in the coastal zone caused by gravity flow adds to the downward propagation of the anticyclonic eddies. This downward motion is compensated for by upward motion in the basin interior. Clearly, the downward propagation of the Bosporus plume is just one element of vertical circulation, albeit a very important one, and cannot be separated from the downwelling in the region of coastal anticyclones and the upwelling in the basin interior. By unifying the downward motions in the coastal zone and the upward motion in the basin interior, mixing regulates the balance between the various dynamic controls. This concept is in line with the theory^9^ linking estuarine exchange flow and mixing, as well as with the classification of semi-enclosed basins comparing mixing versus stratifying agents^10^. In summary, the influx of surface freshwater and inflow from the strait maintain the stability of stratification. Mixing (e.g. associated with wind-driven circulation) tends to deepen the halocline, thereby opposing the dilution of surface waters by rivers. Furthermore, the gravity plume from the strait does not simply propagate through a predefined ambient water column; rather, the water column itself adjusts to the processes that govern the plume's propagation.

**SI-4. The Coupled Mixed Layer-Convective Plume Model**

This model, its physics, numerics, and performance is explained in a previous publication^8^, which provides all the necessary details for interested readers. Here, we will outline the key concept behind the model, which aims to stimulate both the water column and the gravity plume. The model simplifies the basin into two compartments: the main water body, represented as a water column, and the gravity plume. The water column is governed by the same physical principles as most 1D models (see main text), but with one important difference: it communicates with the sinking plume (Figure SI-4.1). At every model level, the plume entrains a certain volume of water from the water column and detrains another volume back. The entrained water volume exceeds the detrained volume, causing the plume to increase in size (note that the upper curved arrows are larger than the lower ones). The entrainment physics adheres to the theories of buoyant plumes. As the depth increases, the plume's salinity decreases and its density approaches that of the surrounding water. The depth of sinking depends strongly on stratification. Conversely, stratification also depends on the interaction between the plume and the surrounding water. This interaction also results in the formation of an upwelling in the model column, which compensates for the downward transport of water by the sinking plume.


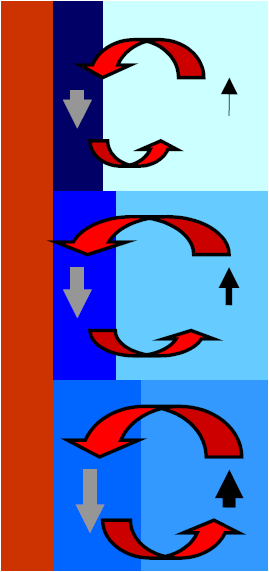


Figure SI-4.1. The concept of a sinking plume^8^. Different shades of blue represent salinity levels (dark blue for high salinity and light blue for low salinity) in a schematic way.

**SI-5. Parameter dependencies in the box model and paleoevolution of the Black Sea as simulated in the 1D plume model**

Table SI-5.1. Dependence of the solution of box-model on the width of strait W for q_R_= 10^4^ $m^{3} s^{-1}$ and h=30 m. The last row shows the fresh-water flux q_R_, for which the ratio $\frac{q_{1}}{q_{2}}=2$. The shaded column (W=950 m) is from the basic experiment discussed in the main text.

| W[m]-> | 500 | 950 | 1000 | 1500 |
| --- | --- | --- | --- | --- |
| $S_{1}$ | 11.3 | 17.9 | 18.4 | 21.8 |
| $S_{2}$ | 16.8 | 21.9 | 22.3 | 25.0 |
| $h_{c}[m]$ | 13.1 | 9.4 | 9.2 | 7.6 |
| $h_{i}[m]$ | 10.9 | 12.6 | 12.7 | 13.3 |
| $q_{1}[m^{3} s^{-1}]$ | 14800 | 20100 | 20700 | 25800 |
| $q_{2}[m^{3} s^{-1}]$ | 4630 | 9980 | 10500 | 15600 |
| $h_{1}[m]$ | 146 | 199 | 204 | 254 |
| $F_{w}[m]$ | 823 | 610 | 594 | 481 |
| $\frac{q_{1}}{q_{2}}=2$ for $q_{R=}X[m^{3} s^{-1}]$ | 5316 | 10127 | 10632 | 15949 |

**SI-6.** **Evolution of the adequacy of modelling strait exchanges in the 3D Black Sea numerical models**

Any long-term simulation of the hydrological state of the Black Sea with a closed strait (i.e. without providing a source of Marmara Sea water) would result in a drift of vertical stratification. Below, we provide a brief overview of the evolution of methods for prescribing or computing the exchange between the Black Sea and the Mediterranean. Some of the earliest consistent 3D numerical models of the Black Sea^11^ accounted for the water and salt balance by ensuring that the total annual mean salinity flux at the sea surface balanced the salinity flux through the Strait of Bosphorus. An experiment carried out with this very early numerical model showed that switching off the river runoff for 34 years only resulted in an almost uniform vertical salinity stratification. Later modelling developments^12^ revealed that a model including part of the Marmara Sea with realistic salinity and a coarse resolution in the strait produced overly large salt transport. To achieve a more realistic salt transport, the authors reduced the contrast between the two basins (reduced salinity of Mediterranean water) and implemented some theoretical considerations about two-layer transport^13^. However, by reducing the contrast between the characteristics of the Mediterranean plume and ambient water, the sinking of the gravity plume became too shallow. The coarse grid resolution of basin-wide numerical models also inhibited the deep propagation of the gravity plume. This prompted to implement gravity plume parameterization^8^. Further improvements to the inter-basin exchange were achieved by linking the two-layer exchange to the dynamics of sea level in the interconnected basins^14^. Recent numerical modelling using unstructured-grid models has enabled processes in the strait to be correctly resolved^15^. However, these types of models are computationally expensive and cannot be run over millennial time periods.

**SI-7. References**

1. Degens, E. T., & Ross, D. A. Chronology of the Black Sea over the last 25000 years, *Chemical Geology*, *10*, 1 – 16 (1972).

2. Myers, P. G., Wielki, C., Goldstein, S. B., & Rohling, E. J.  Hydraulic calculations of postglacial connections between the Mediterranean and the Black Sea. *Marine Geology*, 201 (4), 253-267 (2003).

3. Georgievski, G., & Stanev, E.V. Paleo-evolution of the Black Sea watershed: sea level and water transport through the Bosporus Straits as an indicator of the Lateglacial–Holocene transition. *Climate Dynamics* 26, 631–644 (2006).

4. Ryan, W. B. F., Pitman, W. C. III, Major, C. O., Shimkus, K., Moskalenko, V., Jones, G. A., Dimitrov, P., Görür, N., Sakinc, M., & Yüce, H. An abrupt drowning of the Black Sea shelf. Mar Geol 138(1–2):119–126 (1997).

5. Aksu, A. E., Hiscott, R. N., Mudie, P. J., Rochon, A., Kaminski, M. A., Abrajano. T. & Yasar, D. Persistent Holocene outflow from the Black Sea to the eastern Mediterranean contradicts Noahs flood hypothesis. GSA Today 12(5):4–10 (2002).

6. Lane-Serff, G. F, Rohling, E. J., Bryden, H. L. & Charnock, H. Postglacial connection of the Black Sea to the Mediterranean and its relation to the timing of sapropel formation, *Paleoceanography*, *12*, 169 – 174 (1997).

7. Stanev, E. Trajectories in Oceanography, Springer Nature Switzerland, ISBN:

9783031337208, 3031337204 (2023).

8. Stanev E., Staneva, J., Bullister, J.L., & Murray, J.W. Ventilation of the Black Sea Pycnocline Inferred from Observed and Model Simulated Chlorofluorocarbon Data. *Deep-Sea Research* I.51, 2137-2169 (2004).

9. MacCready, P., Geyer, R. & Burchard, H. Estuarine exchange flow is related to mixing through the salinity variance budget. *J. Phys. Oceanogr.*, 48, 1375–1384, <https://doi.org/10.1175/> JPO-D-17-0266.1 (2018).

10. Valle-Levinson, A. Dynamics-based classification of semienclosed basins. *Regional Studies in Marine Scienc*e, 46(2):101866, DOI: [10.1016/j.rsma.2021.101866](http://dx.doi.org/10.1016/j.rsma.2021.101866) (2021).

11. Stanev, E. V. On the mechanisms of the Black Sea circulation. *Earth-Science Rev.*, 28, 285-319 (1990).

12. Stanev, E. V., Roussenov, V. M., Rachev, N. H., & Staneva, J. V. Sea response to atmospheric variability. Model study for the Black Sea. *J. Mar. Sys.*, 6, 241-267 (1995).

13. Oguz, T., Ozsoy, E., Latif, M. A., Sur, H. I. & Unluata, U. Modeling of hydrographically controlled exchange flow in the Bosphorus Strait. *J. Phys. Oceanogr.*, *20,* 945–965 (1990).

14. Stanev, E. V., & Beckers, J. M. Barotropic and baroclinic oscillations in strongly stratified ocean basins. Numerical study for the Black Sea. *J. Mar. Sys.*, 19, 65-112 (1999).

15. Stanev, E.V., Grashorn, S. & Zhang, Y. J. Cascading ocean basins: numerical simulations of the circulation and interbasin exchange in the Azov-Black-Marmara-Mediterranean Seas system. *Ocean Dynamics*, 67:1003–1025, DOI 10.1007/s10236-017-1071-2 (2017).
